# The Re-integration of College Innovation and Entrepreneurship Education Under Young Entrepreneurs’ Enterprising Spirit and Professional Music Education

**DOI:** 10.3389/fpsyg.2022.892372

**Published:** 2022-06-16

**Authors:** Xiaoyu Qu, Lei Cheng, Ding Cheng, Shanshan Zhang

**Affiliations:** ^1^School of Music and Dance, Qiqihar University, Qiqihar, China; ^2^School of Art, Ludong University, Yantai, China; ^3^College of Music, Sookmyung Women’s University, Seoul, South Korea

**Keywords:** innovation and entrepreneurship education, professional education, reform in education, enterprising spirit, educational integration

## Abstract

The purpose is to coordinate the relationship between innovation and entrepreneurship education (IEE) and professional education. This exploration is based on the entrepreneurial spirit of young entrepreneurs and the re-integration of IEE and music education in colleges. First, the IEE is studied in theory. Then, the basic criteria for integrating IEE and professional education are studied, and 305 students from a music college in Xi’an are taken as the survey sample. The questionnaire is adopted to investigate the current situation of the integration of IEE and professional education. The results show that 52.1% of students believe that IEE is closely related to professional education. In terms of self entrepreneurship awareness, males’ awareness of self entrepreneurship is higher than females’, and the willingness of self entrepreneurship from freshman to senior is 3.1, 15.5, 26.1, and 30.8%, respectively. For the dominant position in the integrated curriculum, 55.6% hold that professional courses should dominate innovative professional courses, and 25.9% believe that innovation and entrepreneurship courses should be dominated. Besides, 16.5% think that the proportion of the two should be the same, and 2% hold that it doesn’t matter. For the enthusiasm of innovative professional courses, only 14.1% of students are very positive. The survey results show that the integration of IEE and professional education needs to be improved, and there is a lack of pertinence and guidance for students of different genders and grades. Students are not clear about the position of IEE and lack enthusiasm. Finally, reasonable suggestions are put forward in view of the above problems. The results are conducive to promoting and accelerating the process of talent training mode combining professional education and IEE. It has a certain reference value for college education and teaching reform.

## Introduction

In the 21st century, innovation has become an inseparable part of people’s life. With the progress of the times and the needs of the practice, the state and society attach great importance to the cultivation of innovative and entrepreneurial talents. As an important part of China’s higher education, colleges undertake a crucial mission in promoting the implementation of innovation and entrepreneurship development strategies, helping to build an innovative country and cultivating high-quality talents for the country (Zhou, 2021). As a training base for high-quality talents, colleges need to adjust their educational policies as soon as possible and coordinate the relationship between innovation and entrepreneurship education (IEE) and professional education. Thereby, how to organically integrate IEE with professional education and make them work together to cultivate students with higher quality and promote full employment and entrepreneurship is an urgent problem in the development of colleges (Huang, 2021).

The innovative function of entrepreneurs plays a very important role in the operation of enterprises, so the entrepreneurial spirit is mainly reflected in the innovative spirit (Wu and Chen, 2021). Based on the innovation-driven development strategy, exploring the combination of IEE and professional education in colleges can scientifically and reasonably integrate IEE into professional courses. Moreover, it can also improve the college education and teaching system reform (Rao, 2020). The purpose of infiltrating and integrating the idea of IEE in professional education is to fundamentally cultivate a group of high-quality professionals with an innovative spirit. It is conducive to determining the direction of college talent training, enriching the talent training mode and theory, and improving the training effectiveness (Ma et al., 2020). The cultivation of innovation and entrepreneurship ability is integrated into professional teaching based on the current integration of college professional education and IEE. Moreover, studying the path of the integration of the two is conducive to optimizing the theory and thought of innovative professional education. It has important theoretical and practical significance for the study of the reform and development of higher education in China.

The purpose is to coordinate the relationship between IEE and professional education, and cultivate more high-quality talents for the country. This exploration is based on the college professional education of music majors. First, the theory of IEE is studied. Then, the basic principles of integrating IEE and professional education are explored. With a music college in Xi’an as the research object, the current situation of the integration of IEE and professional education is investigated through the questionnaire. Finally, the problems reflected in the survey results are analyzed and relevant suggestions are put forward. The research innovation is to promote the theory to practical application through the method of the questionnaire survey, make the reflected problems more specific, and make the follow-up suggestions more targeted. This exploration is conducive to promoting the process of talent training mode combining professional education and IEE, and has a certain reference value for the education and teaching reform of colleges.

## The Integration and Correlation Between Innovation and Entrepreneurship Education and Professional Education

**FIGURE 1:**
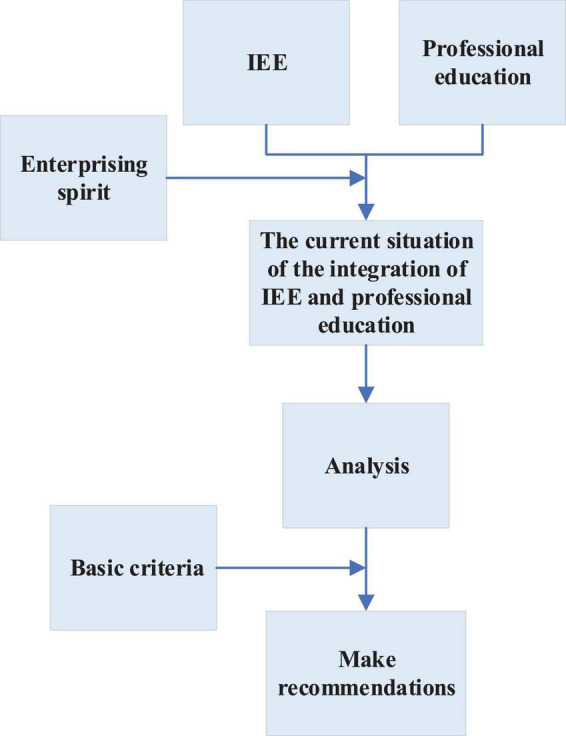
Research framework.

This exploration integrates IEE and professional education to find a more suitable training mode for domestic colleges. Figure 1 is the main framework of this exploration.

This study integrates university IEE with professional education, and adds entrepreneurial spirit in the process of integration, discusses and analyses the current situation of integration education. Additionally, it establishes the basic criteria for the integration of IEE and professional education, and finally puts forward relevant suggestions for the integration education at the current stage through research.

### Enterprising Spirit and Innovation and Entrepreneurship Education

Enterprising spirit is an important and special intangible production factor for entrepreneurs to organize, establish and manage enterprises. For a long time, the concept of the entrepreneur is usually defined from the aspects of business, management and personal characteristics (Chen, 2019). After entering the 20th century, the definition of enterprising spirit has been extended to the fields of behavior, psychology and sociological analysis. In modern western developed countries, it is very common for entrepreneurs to work in government or social organizations, and they also continue to propose and implement the use of enterprising spirit to transform government service and social management (Rogoza et al., 2018; Wu and Song, 2019).

The content of enterprising spirit involves a wide range, which can be summarized into the following four basic aspects. (1) Innovative spirit. Innovation is the soul of entrepreneurs. Compared with ordinary operators, innovation is the main feature of entrepreneurs. The innovative spirit of entrepreneurs is reflected in that a mature entrepreneur can find opportunities that ordinary people cannot find, use resources that ordinary people cannot use, and find methods that ordinary people cannot think of Wu and Wu (2017). (2) Adventurous spirit. To succeed and become an outstanding entrepreneur, a business operator needs to have the spirit of adventure. It is mainly reflected in the formulation and implementation of enterprise strategy, the expansion and reduction of enterprise production capacity, the development and application of new technologies, the opening of new markets and territory, the increase and elimination of production varieties, and the increase or decrease of product prices (Wu et al., 2019). (3) Entrepreneurship. The entrepreneurial spirit of entrepreneurs refers to the spirit of forge ahead, hard work, dedication, diligence and thrift. It is mainly reflected in the positive and enterprising spirit, overcoming the psychology of following the rules and being conservative, the tenacious struggle of entrepreneurs, the professional ethics of dedication and respect for duty, and the spirit of diligence and thrift (Yuan et al., 2020). (4) The spirit of tolerance. The tolerant spirit of entrepreneurs refers to the attitude and spirit that entrepreneurs are tolerant, willing to get along well with others and willing to cooperate with others (Wu W. et al., 2020; Wu Y. J. et al., 2020).

Entrepreneurship education comes from the United States. With the continuous progress of entrepreneurship education, people gradually realize that relying on college students’ entrepreneurship to solve the employment problem is unrealistic, and there are too many limitations in college students’ entrepreneurship (Milligan, 2019). Therefore, scholars integrate the spirit of innovation into entrepreneurship education, so that college entrepreneurship education is no longer equivalent to employment training, but to enable students to achieve more comprehensive development. In 2010, the Ministry of Education of the People’s Republic of China put forward the new concept of “IEE” for the first time, added “innovation” to entrepreneurship education, oriented the related teaching concept and mode to all students, and integrated them into the process of student education and training. The purpose was to improve students’ innovation awareness, entrepreneurship awareness, entrepreneurial ability and sense of social responsibility (Hameed et al., 2021). In 2016, IEE began to be introduced into vocational education. The state vigorously promoted the development of IEE in vocational colleges and specially approved the establishment of 12 “national IEE bases in vocational colleges.” During this period, the focus of college IEE had shifted from entrepreneurship to innovation, which differed a lot from the entrepreneurship education in society to promote people’s employment. The college IEE in this period focused on the theme of promoting the all-round development of students. The educational object changed from a few elites to all students. The scope of education was no longer limited to the campus, and a lot of practical activities were added to promote the all-round development of students’ innovative spirit and entrepreneurial ability (Paul et al., 2021). IEE can effectively improve the comprehensive quality of students. It is an important way for the country to cultivate innovative talents and the development direction of higher education reform in the future.

### Basic Criteria for the Integration of Innovation and Entrepreneurship Education and Professional Education

In addition to long-term exploration and practice and designing a reasonable and scientific integration system, the promotion of the integration of professional education and IEE should also follow the following three principles: adaptability principle, demand-oriented principle, and step-by-step principle (Yang, 2020). Colleges should be aware of their own disciplinary advantages, transform them into IEE, integrate forces to cultivate students’ innovative ability and professional skills, and improve students’ comprehensive quality (Ge and Gao, 2020).

At present, professional education is still the focus of college education in China. Students learn basic professional knowledge and have a strong professional ability, which is the direction of professional talent training in China. Therefore, when integrating professional education and IEE, colleges in China should pay attention to the cultivation of professional talents and systematicness. Meanwhile, they need to cultivate comprehensive entrepreneurship and innovation talents and pay attention to the practicability of education. According to the principle of adaptability, colleges in China should integrate IEE courses when cultivating highly qualified professionals (Qian et al., 2020). In the organic integration of the two, colleges should follow the principle of adaptability from three aspects. First, they should integrate the measures of IEE into the process of professional talent education. While cultivating professional talents, innovative ideas, curriculum setting and credit evaluation should be integrated into the teaching plan. Second, the integration of the two courses is the focus of the practical scheme. Among them, course content, class time and teachers are the necessary conditions for integration. Third, the integration of the two should attach great importance to the practicality of IEE, including the construction of implementation sites inside and outside the school, and the formulation and arrangement of courses (Qian et al., 2018).

Professional education and IEE are two indispensable components of higher education. The cultivation of high-level professionals should pay attention to the education of professional knowledge and cultivate their ability of innovation and the spirit of daring to practice. Therefore, professional education and IEE are indispensable (Lee, 2020). Colleges should follow the demand-oriented principle and stand in the complementarity perspective to make them integrate effectively. First, they should actively look for effective reform plans and innovate talent training strategies. While meeting the professional knowledge training, colleges should scientifically integrate the concept of IEE into the curriculum, coordinate the relationship between students’ professional theoretical knowledge and practical ability training, and effectively improve the teaching quality. Besides, economic development requires more high-level talents with innovation and entrepreneurship ability. This demand requires the organic combination of cultivating students’ innovative spirit and professional theory education, so that the results of IEE can be realized, and the two can work together to improve the overall level of talents (Gao, 2020; Zhu, 2020).

With the in-depth college reforms in China, colleges need to improve the quality of talent training and promote the organic integration of IEE and professional education, which also promotes the pace of college reform. The cultivation of professional talents should pay attention to students’ psychological and physiological changes during their growth. The same is true of IEE. Hence, in the process of college reform, colleges should fully respect the growth and change of students, use the principle of step-by-step, and carry out continuous exploration and practice. Besides, they need to change old ideas, break through inertial thinking mode, and constantly carry out reform and innovation to adapt to the continuous reform of social reality and actively improve students’ professional level and entrepreneurship and innovation ability (Zheng et al., 2018; Zhang et al., 2020).

### The Current Situation of the Integration of Innovation and Entrepreneurship Education and Professional Education

A music college in Xi’an is taken as the research object. It is a college for training music and art professionals, which is one of the 11 independent music colleges in China. There are 580 teaching staff and 5,331 full-time students. There are 10 teaching units, including the composition academy, the college of humanities, the department of piano, the orchestra academy, the national instrumental music academy, the vocal music academy, the contemporary music academy, the music education academy, the college of dance and the college of Marxism.

The form of a questionnaire is adopted in the survey. The contents of the questionnaire are discussed in detail with relevant experts. The questionnaire questions are mainly set for the following five aspects. The first is students’ entrepreneurial intention, the second is students’ views on the integration of the two education, the third is students’ suggestions on the curriculum of innovative professional courses, the fourth is students’ tendency toward the teaching methods of innovative professional courses, and the fifth is to make targeted investigation on the suggestions to improve the integration. This study adopts paper questionnaire, which is randomly distributed to students majoring in music. After that, the SPSS Statistics 25.0 system is used for relevant data statistics and analysis and research. The attached Table 1 presents the specific contents of the questionnaire.

**TABLE 1:** Questionnaire on the integration of innovation and entrepreneurship education (IEE) and professional curriculum education.

Gender	Grades		Major
Single choice questions			
1. What do you think of IEE? ( )			
A. It’s just part of employment education		B. It is mainly entrepreneurship training	
C. It can be conducted separately		D. It should promote each other with professional education	
2. Do you think there is any connection between college professional courses and IEE? ( )			
A. The two are closely related		B. The two have little to do with each other	
C. There is no connection		D. I am not sure	
3. Have you considered starting your own business in this major? ( )			
A. I’ve been planning my own business		B. I thought about it but didn’t implement it	
C. There are students around me who start their own businesses. I want to try it myself		D. I didn’t think about it	
4. What kind of help do you want most now for future entrepreneurship? ( )			
A. Integrated cultivation of entrepreneurship education and professional course education		B. Lectures and counseling related to entrepreneurship	
C. Relevant counseling books		D. I don’t know	
5. Do you think college IEE needs to be integrated with professional education? ( )			
A. Quite necessary		B. Indifferent	
C. Unnecessary		D. I don’t know	
6. Are you interested in the entrepreneurial knowledge involved in professional courses? ( )			
A. Very interested	B. Relatively interested		C. Generally interested
D. Not very interested	E. Completely uninterested		
7. If professional education and IEE are integrated, how should the proportion of courses be allocated? ( )			
A. Focus on professional courses		B. Focus on innovation and entrepreneurship courses	
C. Same proportion		D. Indifferent	
8. How is the IEE carried out around you? ( )			
A. Open elective courses of entrepreneurship education		B. Under the guidance of professional teachers	
C. Carry out entrepreneurship skills improvement training		D. Student organizations promote entrepreneurship education	
9. After the integration of IEE and professional education, how to identify the results? ( )			
A. Theoretical achievements	B. Theoretical score + experimental training score		C. Certificate + professional skills’ entrepreneurship practice
D. Theoretical achievement + experimental training achievement + certificate + professional skills’ entrepreneurial practice			
Multiple-choice questions			
1. What do you think is the main way to integrate the two? ( )			
A. Adopt new teaching materials		B. Adopt information-based teaching	
C. Improve teaching evaluation methods		D. Increase practical teaching	
2. How do you think to promote the combination of professional education and IEE? ( )			
A. Carry out entrepreneurial practice activities	B. Carry out extracurricular entrepreneurial activities		C. Highlight IEE in professional courses
D. carry out school and enterprise cooperation, industry-University-Research integration	E. Optimize the training program of professional talents		
3. What do you think are the difficulties in the combination of IEE and professional education? ( )			
A. Lack of the entrepreneurial teaching team	B. Loose combination		C. Less entrepreneurial communication with enterprises
D. Little entrepreneurial practice provided by the society	E. Students’ low entrepreneurial enthusiasm		
4. What do you think are the effective integration measures? ( )			
A. Revise talent training plan	B. Build a practical training platform		C. Set up a pilot class combining the two
D. Invite successful entrepreneurs to give lectures		E. Slowly infiltrate into professional courses	
F. Others			
5. What kind of teaching methods should be adopted after the integration of the two? ( )			
A. Project guided participatory	B. Group discussion		C. Heuristic interactive
D. Traditional teaching	E. Others		
6. Who should evaluate the teaching of innovative professional courses? ( )			
A. Teacher		B. Student (mutual evaluation)	
C. Enterprise		D. Society	
7. How should schools improve students’ attention to the new curriculum? ( )			
A. Provide corresponding credits	B. Provide funding		C. Provide practical opportunities
D. Teacher encouragement	E. Others		
8. What support is most needed for the integration of IEE and professional education? ( )			
A. School leadership planning	B. Students’ needs		C. Perfect teachers
D. All aspects of financial support	E. Government policy support		F. Social approval degree
G. Others			

The reliability and validity of the questionnaire are analyzed and calculated. The value of the α coefficient in the questionnaire is 0.8, greater than 0.7, with high reliability. The Kaiser–Meyer–Olkin (KMO) value of the questionnaire is 0.777, greater than 0.5. Bartlett’s test value is less than 0.001, indicating that the questionnaire has high validity.

## Research Results and Analysis

### Investigation Results and Analysis

**FIGURE 2:**
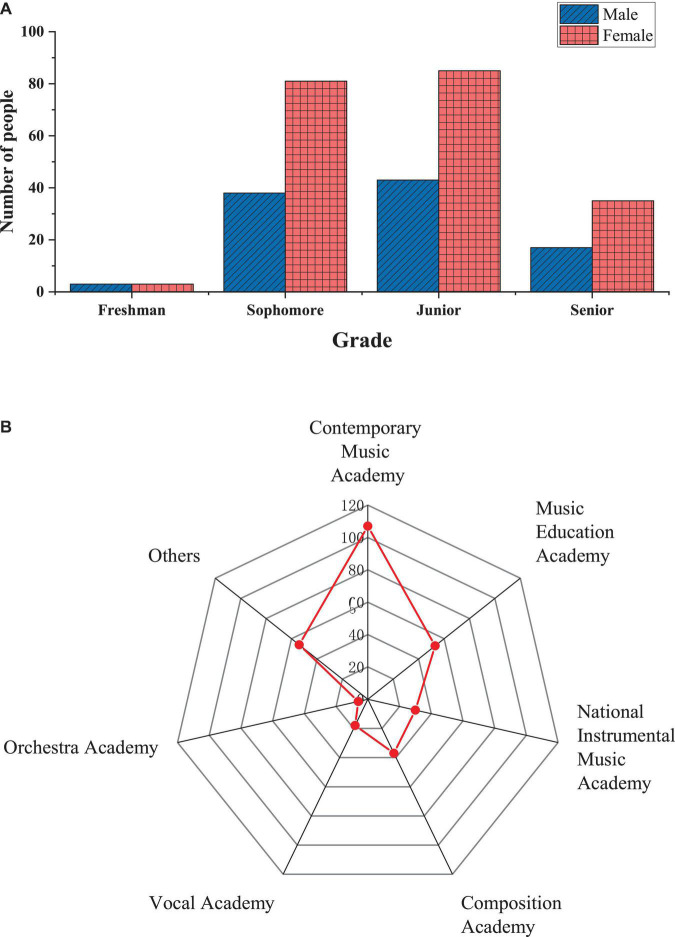
Distribution of sample structure [**(A)** is the grade and gender distribution of the sample, and **(B)** is the major distribution of the sample].

The students surveyed in this questionnaire are all music majors, including 101 males and 204 females. Figure 2 shows the specific sample structure distribution.

Figure 2 shows that among questionnaire participants, 204 are females, accounting for 66.9%, and 101 are males, accounting for 33.1%. 128 are junior students, accounting for 42%, followed by sophomores, 39%, seniors, 17% and freshmen, 2%. In terms of major categories, there are many participants in contemporary music academy, with 107 participants, accounting for 35.1%, followed by other types of students, accounting for 17.7%. The proportion of participants in the music education academy is 17.4%.

**FIGURE 3:**
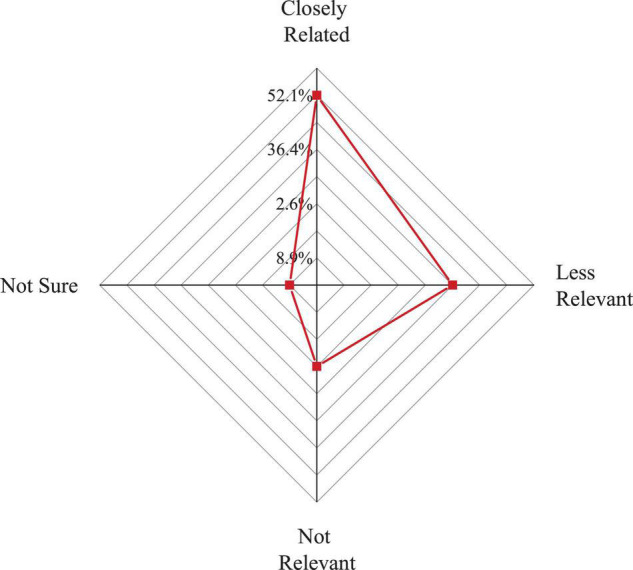
Distribution of students’ cognition of the relevance between IEE and professional education.

Figure 3 shows the distribution of students’ cognition of the relevance between IEE and professional education.

Figure 3 shows that 52.1% of students believe that IEE is closely related to professional education. 36.4% of the respondents hold that there is a relationship between the two, but there is little relationship. 2.6% of students believe that IEE has nothing to do with professional education. 8.9% of the students hold that they cannot explain the relationship between the two. It reveals that in the current education system, although IEE has achieved certain results and can play a positive role in promoting students’ employment and entrepreneurship, it has not been fully integrated with professional education. Students’ positioning of IEE is not clear enough, and their concept of integration of the two is not deep enough.

**FIGURE 4:**
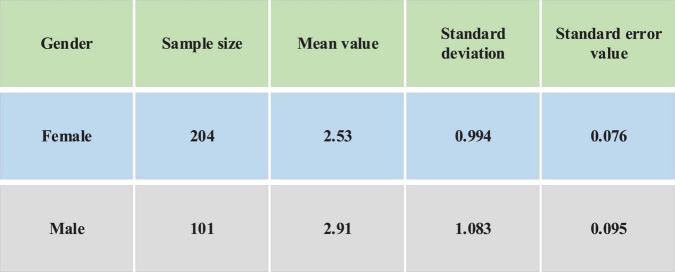
Gender differences in whether students consider starting their own businesses in their major.

Figure 4 shows the gender difference of whether students consider starting their own businesses in their major in terms of the employment prospect of their major.

**FIGURE 5:**
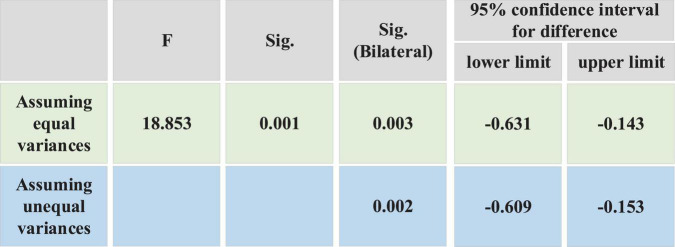
Test results of independent samples.

As Figure 4 shows, there is still a certain gap between the mean values of males and females on whether to consider self-entrepreneurship in this major. The mean value of males is 2.91 and that of females is 2.53. It can be seen that males are more willing to start their own businesses in this major than females. Independent samples are tested. Figure 5 shows the test results.

Figure 5 shows that in terms of the dependent variable of “independent entrepreneurship intention,” the test result is *F* = 18.853 and *p* = 0.001. P reaches the significant level of 0.05, and the alternative hypothesis is accepted. $\sigma_{X\,1}^{2}\neq \sigma_{X\,2}^{2}$ indicates that the two sets of variances are considered unequal. *p* = 0.002 < 0.05, which has reached a significant level of 0.05, indicating a significant difference in the willingness of males and females to start their own businesses. And males’ awareness of independent entrepreneurship is higher than females’ awareness of independent entrepreneurship.

**FIGURE 6:**
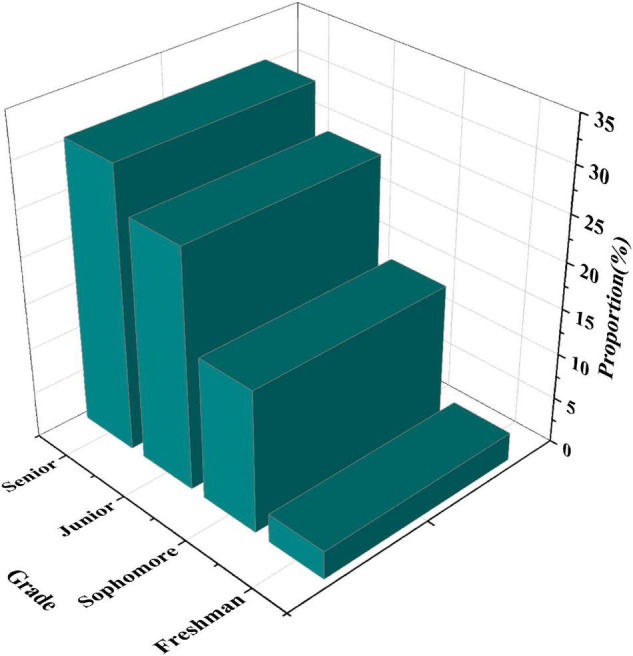
Statistics of the entrepreneurial intention of students in different grades.

Figure 6 shows the statistical results of the entrepreneurial intention of students in different grades.

Figure 6 shows that from freshman to senior, the proportion of students interested in independent entrepreneurship is 3.1, 15.5, 26.1, and 30.8%, respectively. It shows that there are obvious differences in students’ entrepreneurial intentions in different grades. In addition, from the above four data, it can be found that with the increase of grade, students’ entrepreneurial awareness is stronger.

**FIGURE 7:**
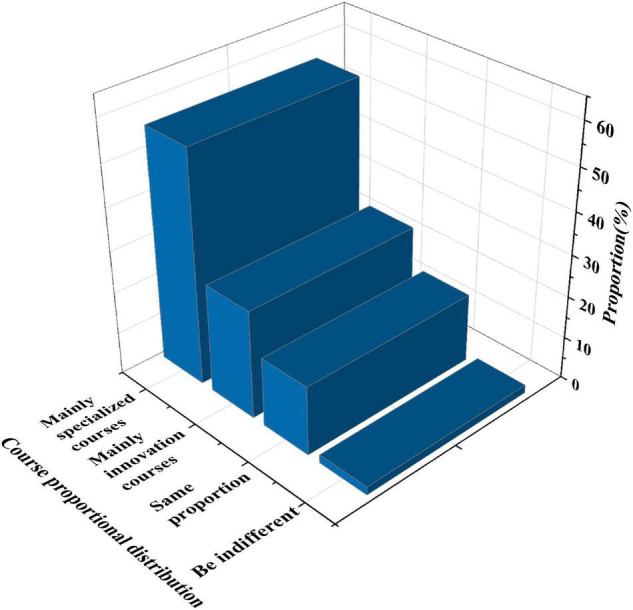
Statistical chart of course allocation proportion.

Figure 7 shows the statistics of course allocation proportion.

Figure 7 reveals that 55.6% of the students believe that the courses integrating IEE and professional education should focus on professional courses. 25.9% of the students believe that the courses should focus on innovation and entrepreneurship. Besides, 16.5% of the students hold that the proportion of the two should be the same. 2% of the students do not care about it. It shows that different students have different requirements for entrepreneurship and innovation courses.

**FIGURE 8:**
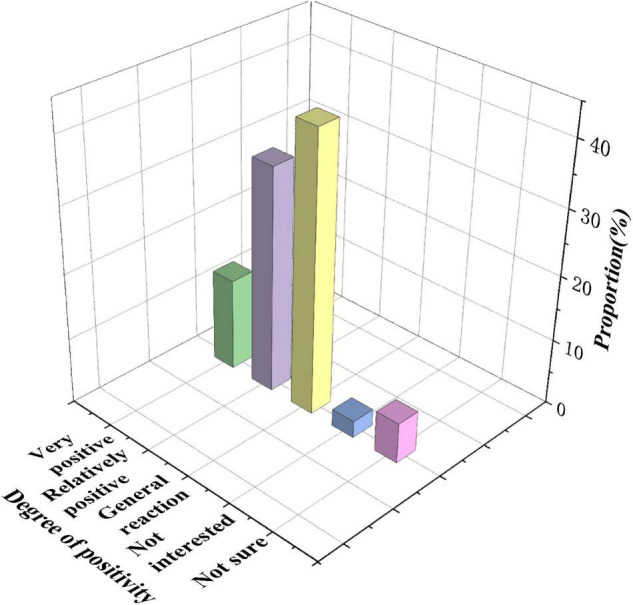
Statistical chart of students’ enthusiasm for entrepreneurship and innovation professional courses.

Figure 8 shows the statistics of students’ enthusiasm for entrepreneurship and innovation professional courses.

Figure 8 reveals that 14.1% of the students have a very positive attitude toward the knowledge of innovation and entrepreneurship involved in professional courses. 34.4% of the students have a relatively positive attitude toward the innovative professional courses. 42.6% show a general reaction, 2.6% are not interested in it, and 6.2% do not pay attention to it and do not know it. The students’ realization of the school’s IEE suggests that the school level advocates the integration of IEE and professional education and actively carries out various activities to promote the deep integration of the two. However, in the actual operation of the integration of the two, students do not actively take the initiative.

### Exploration on the Integration Path of Innovation and Entrepreneurship Education and Professional Education

With the progress of college IEE reform, promoting the integration of entrepreneurship and entrepreneurship education and professional education has become its main task. Based on the survey results and analysis obtained, reference suggestions are put forward for the following aspects:

(1) Strengthen the infiltration of ideas. To deeply integrate IEE with professional education, colleges first need to infiltrate from the concept, and explore and establish a whole process infiltrated entrepreneurship and entrepreneurship education system from the needs of students’ quality improvement and employers’ internal entrepreneurial talents. They need to organically integrate IEE with professional education to enhance students’ innovation awareness and entrepreneurial ability, and cultivate students’ enterprising spirit.
(2) Innovate the curriculum system. Each department of colleges should organize expert teams to develop corresponding courses according to their own subject knowledge characteristics. Colleges should implement the integration of IEE and professional education after considering the characteristics of each academy, each major and each grade. The curriculum content should optimize and integrate multiple disciplines and all kinds of knowledge to form a systematic innovative professional knowledge education system. Colleges should truly integrate IEE with professional education based on the needs of students.
(3) Improve teaching methods. Colleges need to optimize the relationship between theoretical teaching and practical teaching, and unify the traditional professional education practice platform with the IEE practice platform. They can implement interactive classroom teaching reform to promote the interaction between teachers and students and stimulate innovative thinking in classroom teaching. New classroom evaluation standards need to be constructed to promote students’ spontaneous learning and independent thinking. Heuristic and cooperative teaching need to be carried out to encourage and guide teachers of all majors to integrate the cutting-edge academic development of international innovation and entrepreneurship and the latest research results into classroom teaching.
(4) Strengthen school-enterprise cooperation. Colleges should fully use the advantages of enterprises in capital, teachers and production environment, jointly formulate talent training plans with enterprises, and jointly undertake the relevant teaching work of entrepreneurship education. Real projects from enterprises should be introduced. Enterprises should be guided to move the port of employee selection and training to schools. It can provide students with a practical platform and field in an authentic environment. It is also conducive to the timely transformation of scientific and technological achievements and business plans of entrepreneurship education into productivity and economic benefits for enterprises.

## Conclusion

Based on the enterprising spirit of young entrepreneurs, this exploration studies the re-integration relationship between college IEE and professional music education. With 101 males and 204 females from a music college in Xi’an as the survey samples, the current situation of the integration of IEE and professional education is investigated. The survey results show that 52.1% of students believe that IEE is closely related to professional education. For the awareness of independent entrepreneurship, males’ awareness of independent entrepreneurship is higher than females’. Moreover, with the increase of grades, the willingness to start a business will also increase. In addition, 55.6% of the students believe that the Integrating IEE and professional education should focus on professional courses. 25.9% of the students hold that the courses should focus on innovation and entrepreneurship courses. 16.5% of the students think that the proportion of the two should be the same, and 2% of the students hold that it doesn’t matter. Among all the participants, 14.1% show a very positive attitude toward innovative professional courses. 34.4% of the students have a relatively positive attitude. The survey results show that the integration of IEE and professional education is not complete. The education of students of different genders and grades lacks pertinence and guidance. Students are not clear about the position of IEE and lack of enthusiasm. In view of the above problems, this exploration puts forward suggestions from four aspects: teaching ideas, curriculum system, teaching methods, and school-enterprise cooperation. Due to the small number of investigation samples, the reliability of the final results needs to be improved. More relevant investigations will be carried out in the follow-up to increase the research reliability. This exploration has a certain reference value for college education and teaching reform. This exploration can improve the reform of the education and teaching system in colleges in the future and cultivate a group of high-quality professionals with an innovative spirit. It is conducive for colleges to determine the direction of talent training, enrich the talent training mode and theory, and improve the effectiveness of talent training in colleges.

## Data Availability Statement

The raw data supporting the conclusions of this article will be made available by the authors, without undue reservation.

## Ethics Statement

The studies involving human participants were reviewed and approved by the Qiqihar University Ethics Committee. The patients/participants provided their written informed consent to participate in this study. Written informed consent was obtained from the individual(s) for the publication of any potentially identifiable images or data included in this article.

## Author Contributions

All authors listed have made a substantial, direct, and intellectual contribution to the work, and approved it for publication.

## Conflict of Interest

The authors declare that the research was conducted in the absence of any commercial or financial relationships that could be construed as a potential conflict of interest.

## Publisher’s Note

All claims expressed in this article are solely those of the authors and do not necessarily represent those of their affiliated organizations, or those of the publisher, the editors and the reviewers. Any product that may be evaluated in this article, or claim that may be made by its manufacturer, is not guaranteed or endorsed by the publisher.
